# Monochorionic quadramniotic and triamniotic pregnancies following single embryo transfers: two case reports and a review of the literature

**DOI:** 10.1007/s10815-015-0611-2

**Published:** 2015-11-12

**Authors:** Sotirios H. Saravelos, Ting Zhang, Jacqueline Pui Wah Chung, Lu-Ming Sun, Yun Sun, Tin-Chiu Li, Zi-Jiang Chen

**Affiliations:** Assisted Reproductive Technology Unit, Prince of Wales Hospital, The Chinese University of Hong Kong, Shatin, Hong Kong; Center for Reproductive Medicine, Shanghai Key Laboratory for Assisted Reproduction and Reproductive Genetics, Renji Hospital, School of Medicine, Shanghai Jiao Tong University, Shanghai, 200135 China; Fetal Medicine Unit and Prenatal Diagnosis Center, Department of Obstetrics, Shanghai First Maternity and Infant Hospital, Tongji University School of Medicine, Shanghai, China

**Keywords:** IVF, ICSI, Pre-implantation genetic diagnosis, Multiple pregnancy, Ultrasound

## Abstract

**Purpose:**

The purpose of this study is to report two cases of monozygotic quadruplet and triplet pregnancies following single embryo transfer (ET).

**Methods:**

A 29-year-old woman and a 34-year-old woman underwent ART treatment in two affiliated University based ART units. The first woman underwent ICSI with day 3 embryo biopsy for pre-implantation genetic diagnosis (PGD) followed by day 4 transfer, which resulted in a monochorionic quadramniotic (MCQA) quadruplet pregnancy. The second woman underwent conventional IVF with transfer of a single blastocyst, which resulted in a monochorionic triamniotic (MCTA) triplet pregnancy.

**Results:**

The first patient underwent successful selective foetal reduction at 16 + 3 and 17 + 4 weeks of gestation. Two healthy twin girls were delivered by elective caesarean section at 35 + 6 weeks of gestation. The second patient underwent successful selective foetal reduction at 14 + 1 weeks of gestation. The remaining monochorionic diamniotic (MCDA) twins are well at the time of writing this article.

**Conclusions:**

To our knowledge, these cases represent the first case of viable MCQA pregnancy following single ET in the world and the third case of a viable MCTA pregnancy following conventional IVF with single ET. Several factors including blastocyst stage transfer and zona pellucida manipulation have been thought to contribute to monozygotic twinning in the context of ART. These two cases add to the growing literature of monozygotic multiple pregnancies following ART.

## Introduction

The increase of multiple pregnancies has been a major talking point in the field of ART for many years. It concerns not only multiple pregnancies relating to multiple embryo transfers (ET) [1] but also monozygotic twinning relating to single ET [2]. The universal occurrence of monozygotic twining in spontaneous conceptions is estimated to be approximately 0.4–0.45 % [3, 4]. This is traditionally thought to be higher (2–12-fold) in the ART population [5–9], although a recent study from the Danish National Cohort has reported reassuringly similar rates (0.3 %) in both spontaneous and ART derived pregnancies [10].

Higher order monozygotic pregnancies, defined as ≥3 foetuses resulting from a single embryo, are exceedingly rare. For example, the rate of monozygotic triplets, which affects approximately 4.5 % of all triplet gestations, is thought to be less than 0.004 % of all spontaneous conceptions [11–14]. The rate of spontaneous monozygotic quadruplets is even rarer, occurring in approximately 1 in 10–15 million pregnancies [15]. In fact, there have been only a handful of cases reported with ultrasound evidence in the literature worldwide [16–18]. These cases are so rare that there appear to be only 28 cases of documented identical quadruplets in the whole of the USA [15].

As of 2012, over 5 million babies have been born following ART treatment [19]. To date, there appear to be only 18 cases of higher order monozygotic pregnancies reported worldwide in the literature. However, many more may have occurred without been reported in the literature, making the true incidence of higher order monozygotic pregnancies in the ART context difficult to estimate. To our knowledge, there has been only one reported case of monozygotic quadruplet pregnancy following ART treatment, of which all embryos were non-viable at 9 weeks and subsequently were found to have a diagnosis of 46,XX,inv [9] (p11q13) on karyotype analysis [20]. In this article, we report two further cases of high order monozygotic pregnancies following ART: one monochorionic quadramniotic (MCQA) quadruplet pregnancy following ICSI and embryo biopsy for pre-implantation genetic diagnosis (PGD) and one monochorionic triamniotic (MCTA) triplet pregnancy following conventional IVF. To our knowledge, the former is the first case of viable MCQA pregnancy following single ET in the world, while the latter is the third reported case of a viable MCTA pregnancy following conventional IVF with single ET.

## Case 1

A 29-year-old woman with 2 years of primary infertility resulting from male factor infertility (oligoasthenospermia) and abnormal karyotype [45,XY, rob [13, 14] (q10;q10)] underwent PGD-ICSI treatment at the Center for Reproductive Medicine, Renji Hospital, Shanghai. During her first ART treatment cycle, stimulation was performed with daily administration of 150 units of recombinant follicle stimulating hormone (rFSH) (N.V.Organon, Netherlands) according to a standardised long protocol with Triptorelin 0.05 mg daily downregulation (Ferring, Germany). Following hCG trigger, 17 oocytes were collected, of which eight cleavage stage embryos were biopsied. Following fluorescent in situ hybridization (FISH), two normal euploid embryos were diagnosed, of which one was selected for transfer. The embryo selected had undergone single cell biopsy at the 10 cell stage and was subsequently transferred on day 4.

The woman successfully conceived, as confirmed by a hCG level of 2610 IU/L on day 15 post-transfer. Routine two-dimensional (2D) transvaginal ultrasound (US) by a reproductive medicine specialist on day 29 following transfer reported a single gestational sac (37 × 21 × 37 mm) with three yolk sacs and three foetal poles, all with heart beats. The TVU was repeated by the same doctor on day 35 post-transfer reporting a single gestational sac (52 × 19 × 40mm) with three viable foetuses with crown rump lengths (CRL) of 10.5, 8.1 and 10.0 mm. The couple was informed about the increased maternal and foetal risks with this condition and were counselled about the possibility of a foetal reduction procedure.

**Fig. 1:**
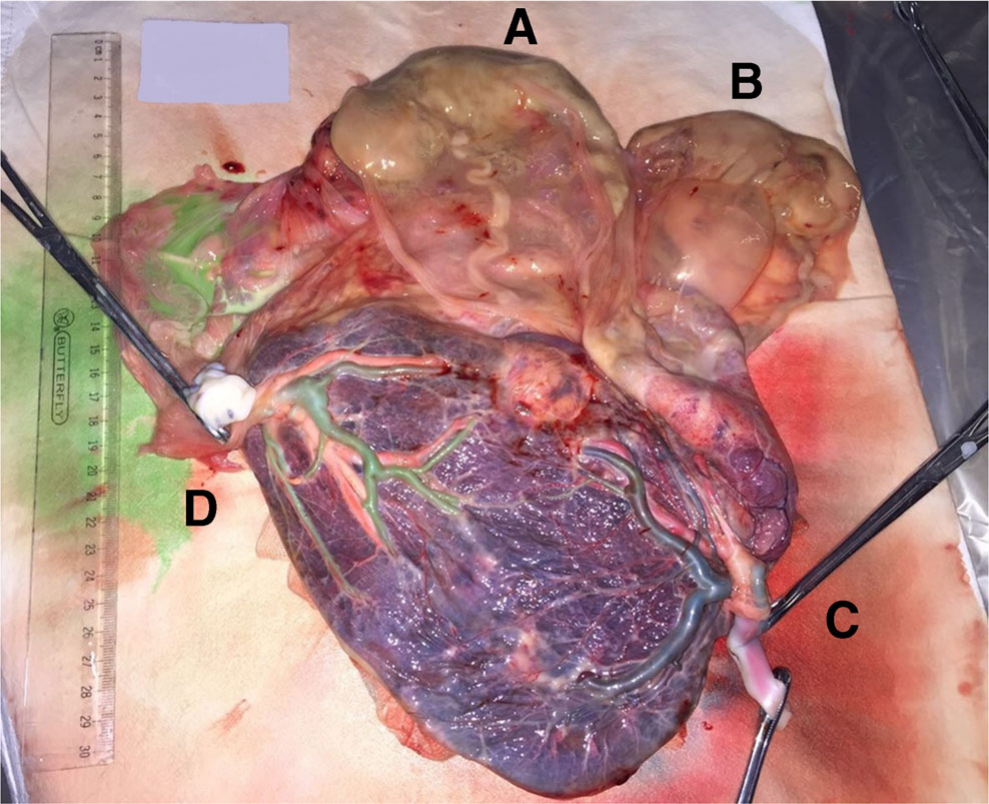
Placental specimen at the time of delivery for case 1 confirming a MCQA pregnancy. **a** and **b** indicate the selectively reduced foetuses, still intact within their amniotic membranes and **c** and **d** the two umbilical cords of the two delivered baby girls. All cords were confirmed to be originating from a single placenta

After consideration, the couple decided to undergo foetal reduction at the prenatal diagnosis centre (PDC) of the First Maternal and Infant Hospital of Shanghai. At 13 + 2 weeks of the pregnancy, an abdominal US was performed by a foetal medicine specialist in fact revealed the presence of a MCQA pregnancy with four equal sized viable foetuses (CRL of 82, 83, 82 and 82 mm), all with nuchal translucency measurements within normal range. The woman subsequently underwent selective foetal reduction via use of radiofrequency ablation on two occasions, at 16 + 3 and 17 + 4 weeks of gestation. In the absence of any definitive evidence, reduction of only one foetus at a time was performed, with the assumption that it may reduce the risk of miscarriage in the remaining foetuses. The procedures were uncomplicated, and two healthy twin girls were delivered by elective Caesarean Section at 35 + 6 weeks of gestation, weighing 2060 and 1985 g. The placental specimen along with the two selectively reduced foetuses confirmed a MCQA pregnancy (Fig. 1).

## Case 2

A 34-year-old lady with 7 years of secondary infertility due to tubal factor underwent conventional IVF treatment at the University ART unit, Prince of Wales Hospital, Hong Kong. She had a previous first trimester miscarriage in 2007 and a right ectopic pregnancy in 2008, at which time she had a salpingectomy. Subsequent investigations including a laparoscopy confirmed blockage of the contralateral tube and evidence of endometriosis. During her first ART cycle, stimulation was performed with daily administration of 225 units of human menopausal gonadotrophin (hMG) (Serono, Aubonne/Switzerland) according to a standardised antagonist protocol with Ganirelix 0.25 mg downregulation (Merck Serono, Germany). Following hCG trigger, 14 mature oocytes were collected, of which 12 were fertilised, yielding 6 viable blastocysts (i.e. Gardner grading of BB grading or above) on day 5. No assisted hatching was performed, and a single expanding blastocyst (i.e. increasing blastocoel cavity with thinning of the zona pellucida and differentiation of the inner cell mass) was transferred to the patient on day 5. The remaining blastocysts were all cryopreserved following appropriate consent.

**Fig. 2:**
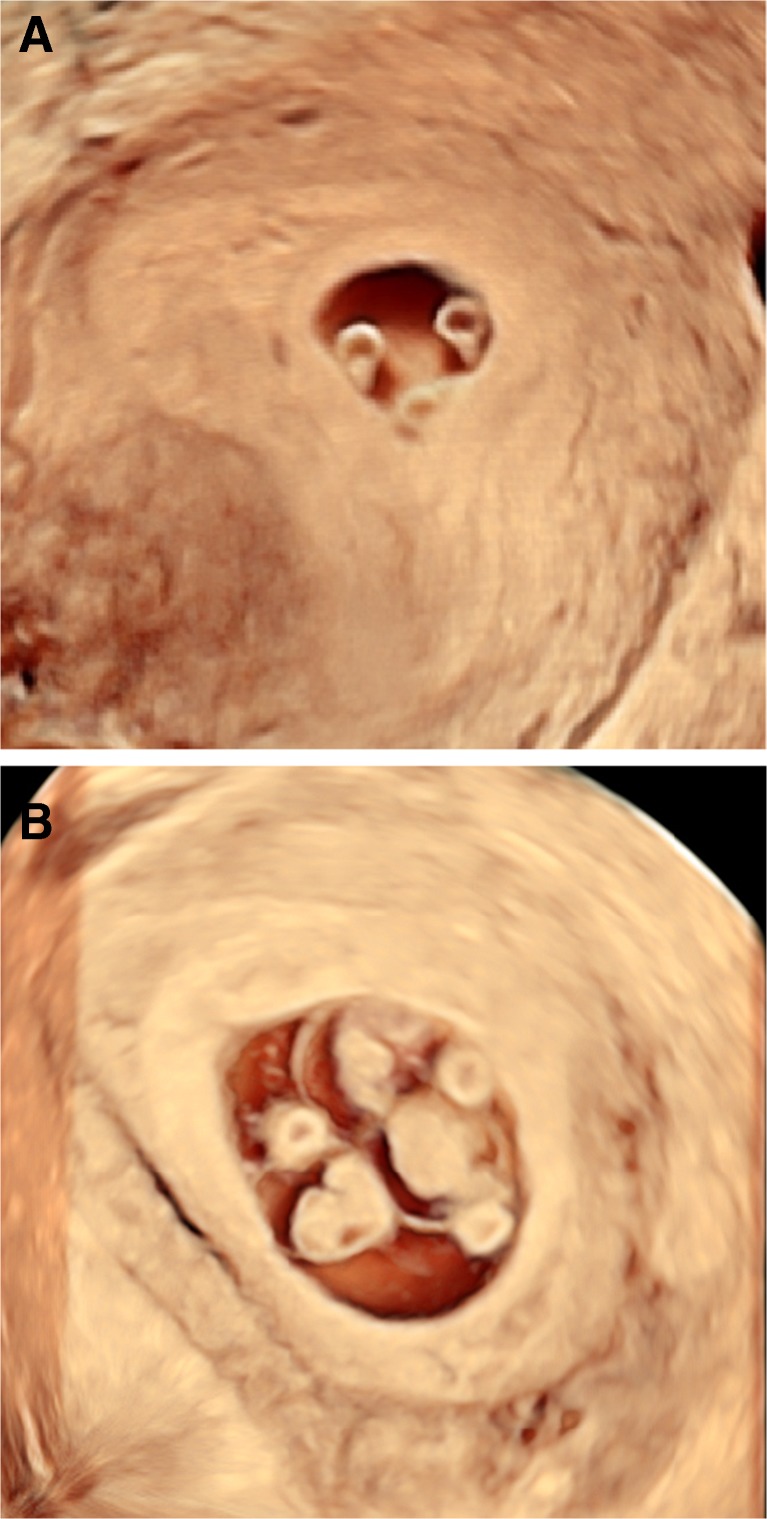
3D US at **a** 23 days and **b** 36 days following transfer clearly demonstrating an MCTA pregnancy

The woman successfully conceived, as confirmed by an hCG level of 154 IU/L on day 9 post-transfer. Routine 2D transvaginal US 23 days following transfer was suspicious of monochorionic twin or triplet pregnancy; therefore, a 3D US was performed on the same day demonstrating clearly a single gestational sac with three yolk sacs and three foetal poles (CRL of 4.5, 3.1 and 3.0 mm) (Fig. 2a). On day 36 following transfer, 3D US confirmed a MCTA pregnancy (Fig. 2b). The couple was informed appropriately regarding the increased maternal and foetal risks of higher order monozygotic gestations and was counselled regarding the possibility of a foetal reduction procedure.

After being reviewed by the foetal medicine team of the Prince of Wales Hospital, an US at 11 + 5 weeks showed all three CRLs of the triplets to be within normal range, although the nuchal translucency was increased in one of the triplets (3.6 mm versus 2.3 and 1.2 for the other two). The couple subsequently decided to proceed with selective foetal reduction via use of radiofrequency ablation, which was successfully performed for the triplet with the thickest nuchal translucency at 14 + 1 weeks of gestation. The procedure was uncomplicated, and the remaining twins are well at the time of writing this article.

## Discussion

To our knowledge, the cases presented in this article represent the first viable MCQA pregnancy in the world resulting from ART and the third viable MCTA pregnancy resulting from conventional IVF with single blastocyst transfer. A systematic review of the literature shows that there are now a total of two monozygotic quadruplets and eighteen monozygotic triplets reported to date worldwide (Table 1). It is interesting to report these two present cases together, as they share many differences. The first case occurred following the transfer of a single day 4 embryo that had been fertilised by ICSI and biopsied for PGD, while the second case occurred following conventional IVF and transfer of a single blastocyst. Both therefore contribute to the growing literature of monozygotic higher order pregnancies following ART.

**Table 1:** Reports of high order (≥3) monochorionic pregnancies following ART in the literature to date

Publication	Zona pellucida breach			Embryonic stage	No of ET	Pregnancies conceived	Intervention	Outcome
	ICSI	Assisted hatching	Embryo biopsy					
Salat-Baroux et al., 1994	No	No	No	Cleavage	4	MCTA triplets	Reduction	DCDA miscarriage
						DCDA twins		
Belaisch-Allart et al., 1995	No	No	No	Cleavage	3	MCTA triplets	Nil	Delivery ?weeks
Yakin et al., 2001	Yes	No	No	Blastocyst	3	MCTA triplets	Reduction	DCDA delivery 36 weeks
						DCDA twins		
Ghulmiyyah et al., 2003	Yes	Yes	No	Blastocyst	2	MCTA triplets	Nil	CS at week 31
Ulug et al., 2004	Yes	Yes	No	Cleavage	3	MCTA triplets	Reduction	Singleton CS at week 38
						Singleton		
	Yes	Yes	No	Cleavage	3	MCTA triplets	Nil	CS at week 34
Unger et al., 2004	Yes	No	No	Blastocyst	2	MCTA triplets	Reduction	Ongoing MCDA pregnancy 22 weeks
						MCDA twins		
Zikopoulos et al., 2004	Yes	No	No	Blastocyst	2	MCTA triplets	Reduction	Ongoing MCDA pregnancy 20 weeks
						MCDA twins		
Risquez et al., 2004	Yes	Yes	No	Cleavage	1	MCTA triplets	Nil	Ongoing pregnancy
								16 weeks
Jain et al., 2004	No	No	No	Blastocyst	2	MCTA triplets	Nil	Ongoing pregnancy 7 weeks
Henne et al., 2005	No	No	No	Blastocyst	2	MCTA triplets	Termination	
Yanaihara et al., 2007	No	No	No	Blastocyst	1	MCTA triplets	Termination	
Lee et al., 2008	Yes	No	No	Blastocyst	1	MCTA triplets	Nil	CS at 33 weeks
Faraj et al., 2008	No	No	No	Blastocyst	1	MCTA triplets	Nil	CS at 32 weeks
Pantos et al., 2004	Yes	Yes	No	Day 4	3	MCTA triplets	Reduction	Singleton CS at 38 weeks
						singleton		
Haimov-Kochman et al., 2009	Yes	No	Yes	Day 4	3	MCTA triplets	Reduction	Singleton delivery at 38 weeks
						singleton		
Liu et al., 2010	No	No	No	Cleavage	2	MCQA	Nil	Miscarriage
Gurunath et al., 2015	No	No	No	Blastocyst	2	MCTA	Termination	
Saravelos et al., 2015	Yes	No	Yes	Day 4	1	MCQA	Reduction	MCDA CS at 35 + 6 weeks
	No	No	No	Blastocyst	1	MCTA	Reduction	Ongoing MCDA pregnancy

*ET* embryos transferred, *CS* caesarean section, *DCDA* dichorionic diamniotic, *MCDA* monochorionic diamniotic, *MCTA* monochorionic triamniotic

Historically, a potential association between monozygotic twinning and zona pellucida structure following ART was first proposed by Edwards et al. (1986) [5]. This was followed by a report of Alikani et al. (1994) on monozygotic twin pregnancies following breaching of the zona pellucida, and the proposal that the zona manipulation may affect the chance of monozygotic twinning [21]. This observation was followed by further publications supporting this hypothesis [22, 23]. The main mechanism suggested for this association is thought to be the herniation of blastomeres through the zona pellucida during blastocyst expansion, which may serve as a trigger for embryo splitting [24]. However, to date, whether this factor is indeed associated with an increased incidence of monozygotic multiple pregnancies remains debatable. Specifically, although there are a number of reports implying a correlation [21–23], others have failed to demonstrate a significant link [25–27]. This has led to some authors concluding that increased reporting of monozygotic twinning in this context may simply reflect the increasing use of procedures involving the zona pellucida in contemporary ART [25]. In our two cases, it is interesting to note that one was a result of ICSI and embryo biopsy for PGD, while the other was a result of conventional IVF with no zona pellucida manipulation. When looking at the higher order monozygotic pregnancies reported to date, 55 % (12/20) were a result of ICSI, 25 % (5/20) had assisted hatching and 10 % (2/20) underwent embryo biopsy. Again, while the numbers are too small to perform meaningful statistical analysis, it is interesting to note that 2/20 have had embryo biopsy, which is generally a relatively uncommon procedure in most ART units.

There have also been several reports suggesting increased risk of monozygotic twinning with embryo transfers during the blastocyst stage [28–31]. Mechanisms suggested for this association involve extended culture time, culture media composition and laboratory experience [32–35]. However, the majority of the reports suggesting this correlation are limited by the fact that they do not include a cleavage stage embryo transfer group for comparison, more recent analyses have somewhat refuted this correlation. A recent meta-analysis found no increased risk in monozygotic twinning in blastocyst versus cleavage stage transfer, when evaluating ART cycles from 2002 onwards [36]. In addition, a study from the Danish National Cohort project found no increase in overall monozygotic twinning rates when comparing women conceiving naturally versus women conceiving following ART treatment, although they did not perform subgroup analysis for the stage of embryo transfer [10]. In our two presented cases, it is interesting to note that one occurred as a result of a day 4 embryo transfer, whereas the other as a result of a blastocyst transfer. Furthermore, when looking at the twenty cases of higher order (≥3) monozygotic multiple pregnancies to date, it appears 55 % (11/20) have occurred as a result of a blastocyst transfer and 45 % (9/20) as a result of cleavage stage transfer (Table 1). Although the numbers are too small to perform meaningful statistical analysis, it can be deduced that currently, there is no significant trend in favour of either stage of embryo transfer.

Another point of interest is that of our two presented cases, the first pregnancy was initially misdiagnosed as a triplet rather than a quadruplet pregnancy until the time when the foetuses were scanned at a later gestational age. In the second case, the diagnosis of twin or triplet pregnancy was suspected at the initial 2D US, but was only clearly confirmed by 3D US, which demonstrated all three yolk sacs and embryos in a single coronal plane. Indeed, making the diagnosis of higher order monozygotic multiple pregnancies with greater confidence at an early stage can be of benefit in the context of ART where the first scan is typically performed at 6 weeks of gestation. This is important as it allows for accurate diagnosis, counselling and referral for appropriate management from very early.

In conclusion, we have presented two cases of monozygotic quadruplet and triplet pregnancies following ART, both of which have undergone successful selective foetal reduction. To our knowledge, the former is the first viable case of monozygotic quadruplet pregnancy in the world, while the latter is the third reported case of a viable MCTA pregnancy following conventional IVF with single ET.
